# *Striga* Biocontrol on a Toothpick: A Readily Deployable and Inexpensive Method for Smallholder Farmers

**DOI:** 10.3389/fpls.2016.01121

**Published:** 2016-08-08

**Authors:** Henry S. Nzioki, Florence Oyosi, Cindy E. Morris, Eylul Kaya, Alice L. Pilgeram, Claire S. Baker, David C. Sands

**Affiliations:** ^1^Kenya Agriculture and Livestock Research OrganizationMachakos, Kenya; ^2^Liberty Initiators NetworkMaseno, Kenya; ^3^Plant Pathology, INRA-PACAAvignon, France; ^4^Department of Plant Sciences and Plant Pathology, Montana State UniversityBozeman, MT, USA; ^5^Biotech InvestmentsBozeman, MT, USA

**Keywords:** *Striga hermonthica*, *Fusarium oxysporum*, Kenya, maize, witchweed, biocontrol, amino acid, toothpick

## Abstract

*Striga hermonthica* (witchweed) is a parasitic weed that attacks and significantly reduces the yields of maize, sorghum, millet, and sugarcane throughout sub-Saharan Africa. Low cost management methods such as hand weeding, short crop rotations, trap cropping, or conventional biocontrol have not been effective. Likewise, *Striga*-tolerant or herbicide-resistant maize cultivars are higher yielding, but are often beyond the economic means of sustenance farmers. The fungal pathogen, *Fusarium oxysporum* f.sp. *strigae*, has been the object of numerous studies to develop *Striga* biocontrol. Under experimental conditions this pathogen can reduce the incidence of *Striga* infestation but field use is not extensive, perhaps because it has not been sufficiently effective in restoring crop yield and reducing the soil *Striga* seed bank. Here we brought together Kenyan and US crop scientists with smallholder farmers to develop and validate an effective biocontrol strategy for management of *Striga* on smallholder farms. Key components of this research project were the following: (1) Development of a two-step method of fungal delivery, including laboratory coating of primary inoculum on toothpicks, followed by on-farm production of secondary field inoculum in boiled rice enabling delivery of vigorous, fresh inoculum directly to the seedbed; (2) Training of smallholder farmers (85% women), to produce the biocontrol agent and incorporate it into their maize plantings in *Striga*-infested soils and collect agronomic data. The field tests expanded from 30 smallholder farmers to a two-season, 500-farmer plot trial including paired plus and minus biocontrol plots with fertilizer and hybrid seed in both plots and; (3) Concerted selection of variants of the pathogen identified for enhanced virulence, as has been demonstrated in other host parasite systems were employed here on *Striga* via pathogen excretion of the amino acids L-leucine and L-tyrosine that are toxic to *Striga* but innocuous to maize. This overall strategy resulted in an average of >50% increased maize yield in the March to June rains season and >40% in the September to December rains season. Integration of this enhanced plant pathogen to *Striga* management in maize can significantly increase the maize yield of smallholder farmers in Kenya.

## Introduction

*Striga hermonthica* (witchweed) is a parasitic weed that attacks cereal crops and significantly reduces yields. Countries with nascent infestation of *Striga* only 25 years ago now have heavy infestations of Striga resulting in significant losses of crop yield, adversely affecting about 300 million people in sub-Saharan Africa. Up to 50 million hectares of croplands in Africa show varying degrees of *Striga* infestation (Ejeta and Gressel, 2007). The most severe impact is made by *S. hermonthica* on maize (Atera et al., 2013). In some locations and years, *Striga* infestation results in total crop failure (Sauerborn, 1991; Abbasher et al., 1998) and abandonment of fields (Debrah, 1994). In Western Kenya alone, *Striga* has infested over 217,000 ha of crop land, resulting in maize losses of 182,227 tons per year valued at 53 million USD (Atera et al., 2013). Recently, *Striga* was reported in the highlands of Kenya^1^. Land use intensification and increasing cereal mono-cropping combined with its quick adaptation to new climatic conditions has exacerbated the *S. hermonthica* problem in sub-Saharan Africa. *Striga* thrives in challenged conditions, including drought and nutrient deficient soils, making it even more detrimental to all farmers but especially smallholder farmers (Ejeta and Gressel, 2007).

Conventional weed management methods including hand pulling emerged *Striga* stalks after they had already damaged the crop, catch/trap crops, crop rotation, and use of tolerant varieties are reported to be not reliably effective against *Striga* (Ogborn, 1987; Atera et al., 2013). There are ongoing efforts to develop maize varieties that are resistant to striga (Badu-Apraku and Yallow, 2009; Venne et al., 2009; Badu-Apraku and Akinwale, 2015) or that are resistant to herbicides that could then be used to control *Striga* (Kanampiu et al., 2003; Ransom et al., 2012). *Striga* tolerant maize varieties generally yield better than susceptible varieties in *Striga*-infested environments; however, their yield is still reduced relative to the yield from non-infested sites (Badu-Apraku and Akinwale, 2015). Such use of hybrid maize germplasm is an effective option for farmers if affordable and available. Farmers need affordable and effective strategies for *Striga* management that can be readily integrated into their production practices (Berner et al., 1996; Ejeta and Gressel, 2007; Hassan et al., 2009). Non-governmental organizations (NGOs) focusing on farm inputs and on education have steered away from investments in farms with *Striga* due to the increased risk of crop failure (Guerena, personal communication 2016; Seward, personal communication 2016), except in cases where they have committed to *Striga* management evaluations, as with the push pull approach with the *Striga* suppressing plant *Desmodium* (Khan et al., 2002).

Biological control of weeds is a promising field with some successes, even though there is an additional complication with biological control of parasitic plants: care must be taken to not harm the host plant. The herbicide industry has confronted the same complication – how to differentially kill one plant and not another. *Fusarium oxysporum* f. sp. s*trigae* has a potential for biocontrol of *Striga* (Ciotola et al., 1995, 2000; Savard et al., 1997; Marley et al., 1999; Venne et al., 2009; Ndambi et al., 2011; Avedi et al., 2014). As a rule, *Fusarium*, mainly *F. oxysporum*, isolated from *S. hermonthica* selectively attack *Striga* spp. (Abbasher and Sauerborn, 1992; Ciotola et al., 1995; Savard et al., 1997; Abbasher et al., 1998; Marley et al., 1999; Amalfitano et al., 2002; Marley and Shebayan, 2005; Elzein and Kroschel, 2006; Schaube et al., 2006; Venne et al., 2009; Watson, 2013). An isolate from Mali in combination with fertilizer inputs successfully prevented *Striga* emergence and resulted in 400% increase of sorghum dry matter (Ciotola et al., 1995). The growth of crop species (sorghum, pearl millet, maize, rice, fonio, cotton, groundnut, cowpea, and okra) was unaffected by this pathogen. However, recent host range studies have shown that some Solanaceous plants are susceptible to *F. oxysporum* isolates used for biocontrol of *Striga* thus they should not be intercropped with *Striga*-host crops where *F. oxysporum* has been applied to control *Striga* (Zarafi et al., 2014). While wild type strains of *F. oxysporum* have been reported to reduce the incidence of *Striga* infestation, their use in the field has not yet been widespread or practical. Perhaps they are not effective enough in the manner applied to restore crop yield or to significantly reduce the soil *Striga* seed bank. Whatever the underlying reasons, biocontrol of *Striga* is not yet used as a routine means of management of this weed and no products are widely available for African smallholder farmers.

The objectives of the work reported here were to validate under realistic field conditions a practical, effective, and inexpensive *Striga* biological control technology that can enable smallholders to greatly improve their economic outlook. Biocontrol agents portend good prospects for effective integration into subsistence farming in particular because of their applicability across a range of crops and varieties, and the minimal economic input. However, there have been several obstacles preventing the widespread acceptance of weed biocontrol practices: (i) cost and availability to the farmer; (ii) sufficient early control of the target weed before it can damage the crop; (iii) reliability of results from season to season; and (iv) greater yields per input of labor. By focusing on these obstacles we developed a labor saving biocontrol of *Striga* technology for rural Kenya and tested it for three seasons in research trials and validated it for two seasons with 500 smallholder farmers. We considered the options of carefully optimizing each variable of our preliminary studies or proceeding to the here-described large scale field testing of our biocontrol strategy in paired plot tests on 500 smallholder farmers’ fields based on preliminary results of the performance of the biocontrol agent. We chose the latter to provide rationale to justify further optimization research.

## Materials and Methods

### Fungal Strains: Isolation and Preservation of *Fusarium oxysporum*

*Fusarium oxysporum* was isolated from wilted diseased *Striga* plants in Western Kenya and identified as described by Nelson et al. (1983). The virulence and host-specificity of the isolates was confirmed in glass house and field studies. The fungal cultures were maintained on PDA (potato dextrose agar (Difco, Detroit, MI, USA)) slants or on sterile wooden toothpicks. Colonized toothpicks were produced by inoculating a plate of PDA with a single culture and incubating for 24–72 h. Sterile toothpicks were then placed directly on the expanding colonies and incubated for a further 72 h. Colonized toothpicks were aseptically removed from the plate and dried in sterile, open glass vials or paper envelopes in a laminar flow hood (24–72 h). The vials or envelopes were then sealed for long-term storage at room temperature (>years). Culture viability and purity after storage was determined by placing a single toothpick on a plate of PDA and incubating for 24–72 h.

### Selection of Strains for Enhanced Virulence to *Striga*

#### Determination of Amino Acid Sensitivity of *S. hermonthica* and Maize in Pot Tests

Amino acid toxicity has long been observed in plants. ‘Frenching’ disease of tobacco, first described in the early 1700’s, is caused by high amounts of isoleucine excreted in the rhizosphere by bacteria (Steinberg, 1952). This amino acid disrupts plant growth and development because of the tight regulation of free amino acid levels in plants (Sands et al., 2003; Sands and Pilgeram, 2009; Pilgeram and Sands, 2010). The amino acids that inhibit *S. hermonthica* but do not inhibit maize were identified using an adaptation of the protocol described by Sands and Pilgeram (2009) in glass house pot tests at KALRO (Kenya Agricultural & Livestock Research Organization) in Kibos. Ten grams of *Striga* seeds that had undergone a dormancy period of more than 4 months were thoroughly mixed with 5 kg of sand. Pots (36 cm diameter) were filled with *Striga*-free black cotton soil. Each pot was infested with *Striga* by mixing one tablespoon of *Striga* -sand mixture (about 20 g) into the soil layer 2 cm below the surface and mixed with the soil. Each pot was then sown with four seeds of a pesticide-free local maize cultivar (cv. Rachari) placed ~2 cm below the soil surface. Five amino-acids (L-lysine, L-leucine, L-tyrosine, L-tryptophan, and L-threonine) were evaluated (5 pots/amino acid). Following planting, pots were watered daily with a solution containing 1 mM of the given amino acid. The control treatment was watered with plain tap water. Watering with amino acids or plain tap water continued until 10 weeks after planting. The trial continued until 15 weeks after planting during which time, both maize and *Striga* had reached maturity. At the end of the trial the dry *Striga* biomass and dry maize biomass were determined.

#### Selection of Amino Acid-Overproducing Variants of *Fusarium oxysporum*

After identifying which amino acids both inhibited *Striga* and did not affect maize (previous step), we selected variants of pathogenic *F. oxysporum* that excreted the selected amino acids. Selection of amino acid-overproducing lines of microorganisms is a relatively straightforward process (Sands and Hankin, 1974; Tiourebaev et al., 2001). The *F. oxysporum* strains were grown in minimal CATSUP media [Czapek’s glucose salts medium (Difco Laboratories, Detroit]) supplemented with thiamine (4 mg/L), proline (2100 mg L^-1^), uracil (20 mg L^-1^), and a commercial vitamin mixture (e.g., Sesame Street Brand, or similar) at 37°C. Amino acid overproducers were selected by plating a suspension of ~10^4^ conidia per dish onto CATSUP agar (2%) supplemented with increasing concentrations (10–1000 mg L^-1^/ml) of an amino acid analog (Sigma–Aldrich, St. Louis). The analogs were: L-3-fluoro-tyrosine for selection of tyrosine excretors, lucinol, or L-norvaline for selection of leucine excretors, and L-seleno-methionine for methionine over-producers. The vast majority of colonies that survived and grew on media supplemented with the toxic analog excreted the desired amino acid which presumably diluted the analog. For example, a colony that grows on media supplemented with 3-fluoro-tyrosine is likely excreting tyrosine. Any colonies growing on media supplemented with an amino acid analog were screened for excretion of the target amino acid using specific amino acid assay media (Difco Laboratories, Detroit) amended with a biological indicator of amino acid excretion [*Pediococcus cerevisiae* (ATTC 4023)]. The relative level of amino acid excretion of selected lines in CATSUP broth was further quantified using HPLC mass spectroscopy. In addition to selecting *Fusarium* variants that excreted amino acids that were toxic to *Striga*, we selected variants of *F. oxysporum* f.sp. *strigae* that excreted the amino acid methionine using the analog seleno-methionine. We chose methionine because it is known to stimulate germination of the *Striga* soil seed bank (Primrose, 1976; Logan and Stewart, 1991) and could enhance the effectiveness of mycelial invasion of the soil seed bank.

### Preparation of T14 Field Inoculum

#### Preparation of Primary Inoculum

Three different amino acid-overproducing variants of *F. oxysporum* were individually cultured on PDA. After the cultures grew for 3–4 days, sterile wooden toothpicks were placed onto the culture. The fungi grew, ramifying into the toothpicks. The toothpicks were removed from the plate and aseptically dried in a laminar flow hood. Once fully dried, the trio of toothpicks was stored together in sealed, sterile drinking straws and transported to the farmers. This 1:1:1 ratio of a trio of toothpicks with variants of *F. oxysporum* f. sp. *strigae* is referred to as Foxy T14.

#### Development and Preparation of Field Inoculum

Kenyan smallholders were given a straw containing the three fungus-carrying toothpicks to inoculate cooked and cooled pearled rice, providing fresh on-farm inoculum of the fungi. In an alcohol-swabbed disinfected plastic container with a lid, the on-farm inoculum was prepared by room temperature incubation of the toothpicks in pearled rice. Although other grains were also tested as substrates, the pearled rice consistently supported abundant mycelial growth if kept unopened and shaken twice daily for a 3-day period. We determined that a period of 3 days of incubation at room temperature was optimal to avoid depletion of the carbohydrates in the inoculum as well as risk of external contamination. The Foxy T14 field inoculum contained all three variant strains of *F. oxysporum* that each excreted tyrosine, leucine and/or methionine.

### Paired-Plot Field Trials on 500 Smallholder Farms

After first successfully testing the method on 50 farms (data not shown), we embarked on a large scale field trial. In 2014 we evaluated Foxy T14 on over 500 smallholder farms near Maseno, Kenya. The 500 trial farms were in a 127 km^2^ region bounded by latitudes 0.059°N and 0.057°S and longitudes 34.588°E and 34.503°E. A survey was first conducted on all of the farms to determine that *Striga* was present and was an apparent constraint to maize production Training of the farmers to deploy Foxy T14 and collect data was coordinated by the association called Liberty Initiators Network (LIN) based in Maseno. Twenty seven LIN farmers, who had previously collaborated with us to evaluate the technology on KALRO research farms, were further trained as implementers to instruct 500 smallholder farmers to establish paired 4.5 m × 10 m plots on their farms. On each farm the Foxy T14 treatment was compared to a standardized farmer-practice. Both treatments used the same hybrid maize, fertilizer, and plot management. LIN purchased maize seed Western Seed cultivar Western 302, untreated with fungicide and fertilizer in bulk for distribution to LIN members. At planting, DAP fertilizer was applied at a rate of 175 kg/ha (80.5 kg P_2_0_5_) ha^-1^. After thinning, CAN fertilizer was top dressed at a rate of 150 kg/ha (40.5 kg N ha^-1^). The amount of fertilizer applied to each hill was approximately 4.4 and 5.3 g of DAP and CAN, respectively. The Foxy T14 treatment was applied as taught to the smallholder farmers by the implementers. As maize is planted by hand by these smallholder farmers, approximately 2.5 g of inoculated rice was placed in each planting hole along with fertilizer and 2 kernels of maize seed (**Supplementary Figure S1**). Each hill was later thinned to a single plant. *Striga* plants were not culled from neither the farmer practice plots nor the T14 plots and were recorded along with dry grain yield at harvest. Manure was applied at 22 g/hill (10 tons per hectare), far below the KALRO’s recommended rate of 100 tons per hectare to have an effect on Striga. A few smallholders did not fertilize the paired plots equally due to an inadequate supply of manure. Their yield data were excluded from analysis. The LIN implementers collaborated with the farmers to collect biweekly readings of the number of emerged and wilting *Striga* plants. The trial was conducted during the March – June rains season and repeated during the September–December rains season.

### Verification of the Absence of *Fusarium* Toxin Production by the T14 Biocontrol Lines of *F. oxysporum* fsp *strigae*

The three enhanced Foxy T14 lines and a wild type line were grown to exhaustion on boiled rice. The colonized rice cultures were tested at The Mycotoxin Laboratory at Virginia Polytechnic University (Niki McMaster and David Schmale, III) using certified GC-Mass Spectroscopy detection methods for five of the common *Fusarium* elicited toxins: deoxynivalenol (DON), 3-acetyldeoxynivalenol (3-ADON), 15-acetyldeoxynivalenol (15-ADON), nivalenol (NIV), and zearalenone (ZEA). None were detected in the Foxy 14 lines nor from a wildtype strain of *F. oxysporum* f. sp. *strigae* also from Kenya. In a separate assay procedure [Veratox-Fumonisin test 5/10, Neogen Corp. Lansing Michigan, Limit of detection: 0.2 ppm (determined by the mean average of 10 fumonisin free samples plus 2 standard deviations) and the limit of quantitation: 0.5 ppm (described as the lowest concentration point on the calibration curve that this test can reliably detect fumonisin)], they tested for the presence of fumonisins (Khatibi et al., 2014). A reference sample of dried distiller grains was concurrently analyzed as a positive control.

## Results

### Screening Amino Acid Sensitivity of *Striga* and Maize Reveals an Amino Acid Cocktail for Virulence Enhancement of *F. oxysporum*

**FIGURE 1:**
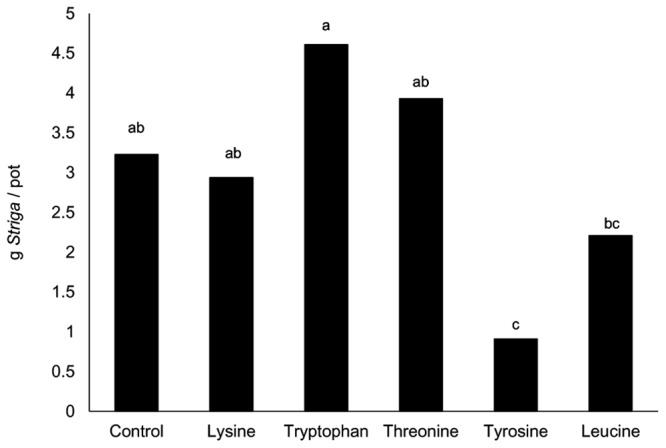
**Effect of an excess of amino acid on *Striga* biomass 15 weeks after planting (WAP) in *Striga* infested maize in pots at KALRO, Kibos.** Means followed by the same letter are not significantly different at *P* = 0.05.

None of the tested amino acids were inhibitory to the maize. In all the treatments, *Striga* emerged 5 weeks after planting. There were apparent trends but with no significant differences (*P* > 0.05) in the number of emerged *Striga* plants, the number of wilting *Striga* plants, or flower/seed capsule formation (data not shown). The treatments varied significantly in *Striga* dry weight (**Figure 1**). Tyrosine resulted in the lowest dry *Striga* weight followed by leucine and lysine. The *Striga* dry weight was highest in the control and the tryptophan treatments. There were no significant differences in maize stover dry weight among all the treatments (data not shown). Based on these results, tyrosine and leucine were the amino acids chosen for virulence enhancement studies. We also selected for methionine over-production in our biocontrol mix (Foxy T14) because methionine is converted into ethylene by soil microbes and ethylene stimulates *Striga* seed germination (Primrose, 1976; Logan and Stewart, 1991) which would render the *Striga* more susceptible to *F. oxysporum.*

Based on the amino acid sensitivity studies, we selected variants of the wild type isolate (Ken Foxy 1/KSM1) that overproduced tyrosine, leucine, and or methionine. We pooled three strains (Leu2a, Z6a and Z5a) that in combination produce excess leucine, methionine, and tyrosine as shown by GC-Mass Spectroscopy analysis of CATSUP broth grown cultures of *F. oxysporum* (**Table 1**). The amounts of production of these amino acids in **Table 1** are only relative to a control wild type strain in *in vitro* culture and are not necessarily indicative of what might be produced by the three strains in the soil or plant tissue.

**Table 1:** Relative enhancement of amino acid excretion in minimal medium (% of total amino acids excreted minus excretion by wildtype).

Amino acid	Strain Leu2a	Strain Z6a	Strain Z5a
Tyrosine	0	0.3	0
Leucine	0.3	4.0	0.2
Methionine	0.1	0.2	0.1

*Foxy T14 inoculum was a 1:1:1 combination of the three strains below*.

### Significant and Consistent Yield Increases were Achieved in On-Farm Testing of Foxy T14

**FIGURE 2:**
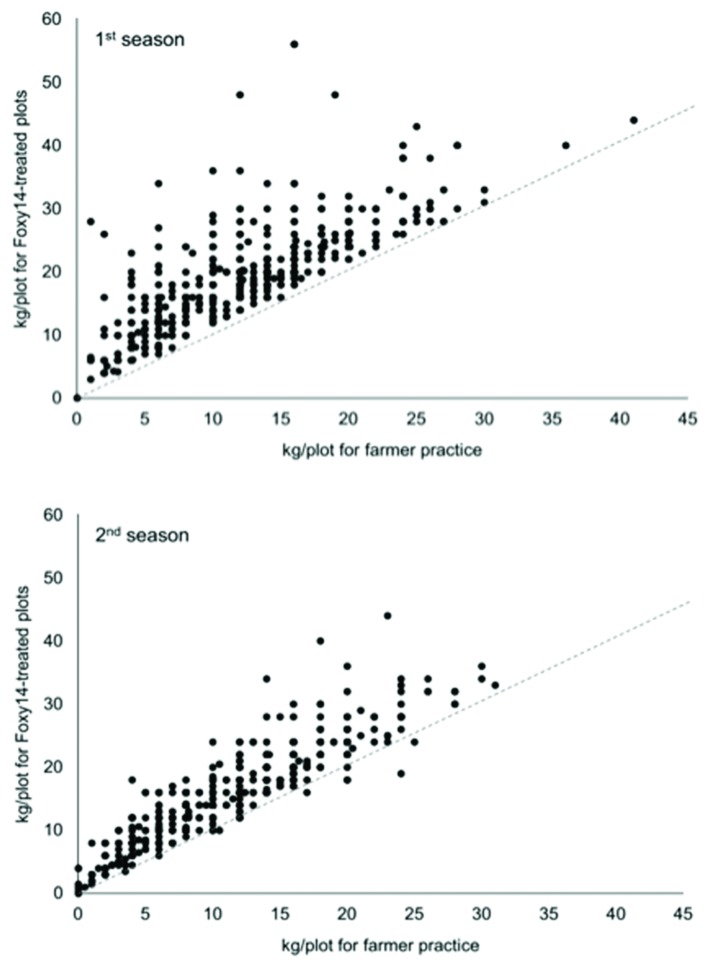
**Maize yield (2014) in the long rainy-season (first season) and in the short rainy season (second season) for Foxy T14 plots relative to maize yield in the corresponding farmer-practice plots (i.e., control).** The average maize yield in the long rainy season increased by 56.5%. Average yield improvement of short rainy season was 42%. Numerous values are super-imposed. The light gray dotted line indicates yields for Foxy T14 plots that are equal (1:1) to that in farmer-practice plots.

The implementers each trained approximately 20 participating farmers on secondary inoculum production and Foxy T14 application. The implementers monitored the fields and collected all the data. Data on 500 *Striga* infested farms were obtained for both growing seasons with almost all the farmers participating in both seasons. Most (99.6%) of the farmers had equal or greater yield in their Foxy T14 plots relative to yield in their comparable farmer-practice plots without Foxy T14 (**Figure 2**). The average maize yield in the March–June rains season was increased by 56.5% in Foxy T14 plots relative to the farmer-practice plots (*p* < 0.0001, pair-wise *t*-test; **Figure 2**). Approximately one third of the farmers doubled their yield in this test (**Figure 2**). Overall, yield in the September–December rains season was reduced by drought but we still observed an average increase of yield of 42% (*t* < 0.0001; **Figure 2**). Typical of maize smallholder farmers in Western Kenya, 85% of the farmers were women. There was no significant linkage between gender and yield.

**FIGURE 3:**
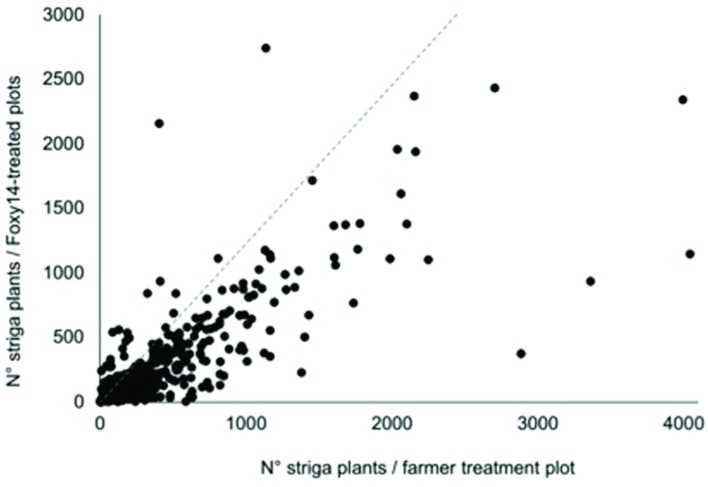
**Number of emerged *Striga* plants in the long rainy -season (first season) for Foxy T14 plots relative to the number in the corresponding Farmer-practice plots (i.e., control) at harvest (14 weeks after planting).** The light gray dotted line indicates *Striga* counts for Foxy T14 plots that are equal (1:1) to those in Farmer-practice plots. The number of *Striga* plants was reduced in 80% of long rainy season Foxy T14 plots.

*Striga* stalk emergence was reduced in 80% of long season Foxy T14 plots and in 92% of the short season Foxy T14 plots (**Figure 3**). Maize yield increases were positively correlated with inherent yield capacity of fields, negatively with above-ground *Striga* density, and positively with the magnitude of reduction of above-ground *Striga* (**Supplementary Figure S2**).

### T14 Biocontrol Lines of *F. oxysporum* Do Not Produce Detectable Levels of Fusarial Toxins

No traces of any of the five common *Fusarium* produced toxins [deoxynivalenol (DON), 3-acetyldeoxynivalenol (3-ADON), 15-acetyldeoxynivalenol (15-ADON), nivalenol (NIV) and zearalenone (ZEA)] were detected in the Foxy 14 lines nor from a wildtype strain of *F. oxysporum* f. sp. s*trigae* also from Kenya. Additionally, Niki McMaster and David Schmale, conducted a separate analysis, in which additional toxins (fumonisins) were also not detected in any of the samples.

## Discussion

Our work clearly shows that deployment in the field of actively growing strains of *F. oxysporum* pathogenic to *S. hermonthica* and selected for over-excretion of amino acids that are inhibitory to this parasitic weed can significantly increase the yields for smallholder farmers under real production conditions in Kenya. The marked effectiveness of this method provides justification for more in depth molecular studies to assess the precise dynamics of amino acid excretion *in situ* and the extent to which it harms *Striga* development. The complexity and expense of such studies could have hindered by several years the transfer of this technology to the field. In this light, we recognize that, although we have demonstrated the effectiveness of the Foxy14 for biocontrol of *Striga*, the precise mechanisms of its effectiveness remain to be fully clarified.

These results are significant for several reasons. Firstly, yield increase was obtained under all conditions tested including different production seasons and different yield potentials of farms and farmers. Secondly, the technology was readily deployable in that it was correctly implemented by 500 smallholder farmers and is compatible with their economic limitations. Most biocontrol agents are produced in large facilities and then packaged for long term storage for later farm distribution and not optimal for delivery of fresh rapidly growing inoculum. These biocontrol agents are not efficacious in the case in sub-Saharan Africa and Asia where operating farms of 10 hectares or less are frequently vulnerable points in supply chains. The Foxy T14 on-farm inoculum method is ideal for sustainable deployment of biocontrol of *Striga*. Thirdly, by aiding farmers to reliably achieve yields that allow for economic success, this biocontrol technology will better allow smallholder farmers to invest in inputs such as high quality seed and fertilizers.

The use of various forma speciales of *F. oxysporum* for biological control of weeds and in particular of parasitic weeds such as *Striga* has faced numerous obstacles: (1) Plant pathogenic fungi, especially host specific types, are seldom sufficiently lethal to reduce their target weed to the “knockdown” levels that synthetic herbicides can give. (2) Parasitic weeds are especially difficult to control because the control action must not harm the host crop. Fungi have pathological traits including toxin production and hormone actions that can potentially affect the host crop or the farmer. (3) A single *Striga* plant can produce up to 500,000 seeds in a season and these seeds can survive in the soil seed bank for over a decade (Berner et al., 1995). (4) The traditional delivery systems for biological agents involve field inoculum being manufactured in large central facilities and distributed to farms. The inoculum in such formulations has been stabilized (dried) to maximize shelf life and transportation durability. Such biocontrol agents are essentially dormant when they are applied to the soil and take considerable lag time and optimal conditions to ensure maximum viability. They must quickly encounter and infect a host or die from lack of nutrition. By placing the fungus in rice, it has a source of nutrition for a prolonged period, and allows long distance mycelial growth, increasing the likelihood of encountering a germinated *Striga* seed. Once the scientific development costs of centrally produced commercial biocontrol agents are factored into the product cost, they are beyond the means of the primary clients, smallholder farmers. (5) Biological controls, to be truly effective need to be integrated into overall crop management strategies which may include input investments of labor, soil amendments and quality seed. These investments are not justified if *Striga* is present. (6) Biocontrol agents, even if they are isolated locally, face expensive requirements involved in registration of pesticides, including toxicological, environmental, and efficacy studies. (7) Biocontrol is not a silver bullet that will miraculously maximize crop yield, but it can act as an organizing driver for communities to further impact their wellbeing. This was observed by their continued participation of the women in the Liberty Initiators Network.

Once a problematic weed is suppressed, crop yield is still influenced by the quality of seed, soil fertility, and water availability. When the above obstacles are overcome, there is still the challenge of getting the technology into the hands of the smallholder farmer who is simultaneously battling poor nutrition, disease, grain storage problems, illiteracy and lack of education, gender inequality and poverty. The successful biocontrol of *Striga* can have a strong impact when integrated into a rural community, not only for its direct economic impact, but it also strongly contributes to womens’ empowerment, rural nutrition, and education for children. With effective *Striga* management, smallholder farmers can reduce the risk of crop failure and enabling them to improve their outlook by adopting a series of agronomic practices. Effective *Striga* management clearly and concretely contributes to sustainable food security for these farmers.

The method of biocontrol of weeds described here is also compatible with farming systems based on agroecology. The amino acid excretion approach to possible virulence enhancement does not involve directly amplifying plant hormones or phytotoxins. Since selections of amino acid excretion are commonly done by industry to produce large amounts of amino acids for animal feed and even for human nutritional supplementation, the choice of this enhancement method was based on safety considerations relative to the selection of toxin-producing strains. Numerous countries are attempting to reduce the use of and exposure to chemical pesticides, however, replacement strategies are not emerging quickly. The approach demonstrated here of selecting for enhanced virulent biocontrol agents may be applicable to many weeds in that amino acid sensitivity of plants is common, as are host-specific pathogens that could be enhanced for virulence in this manner. We should note that there are rare cases where phenylalanine, an essential amino acid, can have negative effects on humans.

The virulence enhancement and extensive field trials of Foxy T14 led to a replicable technology that provides consistent reduction of *Striga* paired with increased maize yield on smallholder farms. In the hands of farmers, this technology increased maize yield by 56.5% in the long rains season and by 42% in the short rains season. After limiting the effect of *Striga* in the paired plot experiments, there were still great two–fourfold differences in yield among the 500 farms. These differences can probably be attributed to multiple factors including farmer agronomic knowledge, planting date, drought, flooding, and soil nutrient deficiency. We speculate that soil nutrient deficiency, particularly of trace minerals not addressed with the DAP and CAN input used in the paired plot trials, is a principal compounding factor. Trace element deficiency could not only impact crop yields but, as a consequence of a mineral deficient diet, could also have a negative impact on smallholder health with increased incidence of malaria and cognitive disorders.

## Conclusion

Field deployment of strains of *F. oxysporum*, inherently pathogenic to *S. hermonthica* and then selected for their excretion of certain amino acids, could significantly increase maize yield of smallholder farmers under real production conditions in Kenya. The results are significant for several reasons. Significant yield increases were obtained under all conditions tested including different production seasons and different yield potentials of farms and farmers. The technology was easily and eagerly implemented by 500 smallholder farmers. This technology is inexpensive to manufacture, making it an affordable investment for smallholder farmers. The toothpick primary inoculum system is stable, inherently inexpensive, easily distributed, and it was embraced as part of the on-farm culture where cooking is done daily. By aiding farmers to curtail *Striga* and reliably achieve yields, this biocontrol technology could enable smallholder farmers to purchase inputs such as high quality seed and fertilizers, understanding that their investment will not be rendered futile by *Striga* infestation.

Endemic pathogens may be best adapted to local conditions, and they may present less risk from unforeseen side effects if toxigenic strains of *Fusarium* are avoided. This technology of strain enhancement relies on concerted conventional selection of mutations in amino acid biosynthesis pathways. We chose to develop a three component enhancement of virulence to increase the field effectiveness of the biocontrol treatment and to hopefully delay resistance as is common in weeds when exposed to single herbicides. We expect that this inherently accessible and sustainable low input technology can be incorporated into integrated pest management systems, promoting reduced herbicide and toxin exposure, and, through increased yield income, allow for optimization of plant germplasm diversity and quality.

We envision that this biological control of *Striga* could be adopted in each country where there are *Striga* infestations.

## Author Contributions

HN, FO, and DS designed the study. HN and FO collected the data. HN, DS, CM, EK, AP, and CB analyzed the data. HN, DS, CM, AP, and CB interpreted the data. HN, DS, CM, AP, and CB drafted the manuscript and critically revised the manuscript. All authors read and approved the final version of the manuscript.

## Conflict of Interest Statement

The authors declare that the research was conducted in the absence of any commercial or financial relationships that could be construed as a potential conflict of interest.

## Footnotes

1 http://striga.aatf-africa.org

## Supplementary Material

FIGURE S1 **Smallholder farmers apply the inoculated rice while planting**.

FIGURE S2 **Trial plots: left plot used hybrid seed, fertilizer, and manure**. Middle plot used Foxy T14, hybrid seed, fertilizer and manure. Right third plot used farmer seed saved from a previous harvest.
