# Supplementary figures and images for: Striga Biocontrol on a Toothpick: A Readily Deployable and Inexpensive Method for Smallholder Farmers
Source: Front Plant Sci. 2016 Aug 8;7:1121. doi: 10.3389/fpls.2016.01121 (PMC4976096; doi:10.3389/fpls.2016.01121)

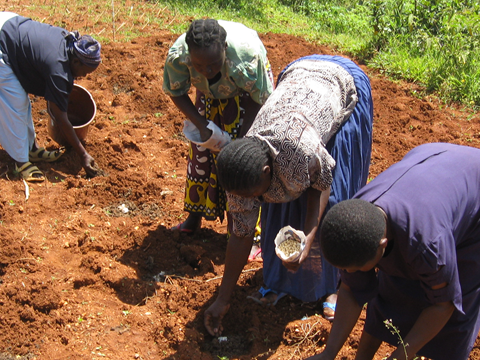

Supplement: FIGURE S1 — Smallholder farmers apply the inoculated rice while planting. [file Image_1.PNG]

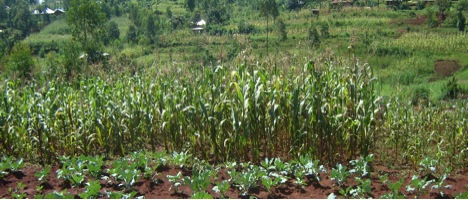

Supplement: FIGURE S2 — Trial plots: left plot used hybrid seed, fertilizer, and manure. Middle plot used Foxy T14, hybrid seed, fertilizer and manure. Right third plot used farmer seed saved from a previous harvest. [file Image_2.JPEG]
